#  Bacterial Contamination of Single- and Multiple-Dose Vials after Multiple Use and Intravenous Admixtures in Three Different Hospitals in Iran

**Published:** 2013

**Authors:** Hossein Khalili, Mehdi Sheikhbabayi, Nasser Samadi, Hossein Jamalifar, Dina Dalili, Nasrin Samadi

**Affiliations:** a *Department of Pharmacotherapy, Faculty of Pharmacy, Tehran University of Medical Sciences, Tehran, Iran.*; b *Pharmaceutical Quality Assurance Research Center, Tehran University of Medical Sciences, Tehran, Iran.*; c *Department of Drug and Food Control, Faculty of Pharmacy, Tehran University of Medical Sciences, Tehran, Iran. *

**Keywords:** Microbial contamination, MDVs, ADXs, Hospitals

## Abstract

There is possibility of microbial contamination of any single-dose vials (SDVs), multiple-dose vials (MDVs) and admixtures (ADXs) during the preparation and injection to the patients that could be resulted in bloodstream infection. The goal of this study was to investigate the microbial contamination of MDVs and SDVs after multiple use and ADXs prepared by nursing staff in the treatment room versus those prepared by the hospital pharmacist in the clean room. The sterility of 43 opened MDVs and SDVs, 92 prepared ADXs in treatment room and 17 prepared ADXs in clean room were studied by membrane filtration method. Only one of 92 ADXs prepared in treatment room was contaminated with *Bacillus subtilis *(%1.1) and none of the ADXs prepared in clean room, MDVs and SDVs had microbial contamination. Although good sanitization practices and training of nurses could reduce the risk of microbial contamination in traditional units, using clean room for preparation of parenteral products could be the best strategy.

## Introduction

Multiple-dose vial (MDV) describes a vial in which antibacterial preservatives are present and may be used more than once based on the manufacturer’s recommendations (1). Single-dose vial (SDV) is intended to be used only once. Admixture (ADX) is a mixture of two or more injection medications. There is possibility of microbial contamination of any SDVs, MDVs and ADXs during the preparation and injection to the patients that could be resulted in septicemia. It has been demonstrated that potentially pathogenic microorganisms can survive and sometimes proliferate in MDVs, which in turn creates a potential risk for parenteral inoculation of pathogenic organisms (1, 2). The rate of ADXs and MDVs contamination was reported in range of 0-14.5% (3) and 0-27% (4), respectively. 

Factors that might affect the quality and sterility of the medication are as follows: number of withdrawals made from the vial, sterility of the techniques employed by the personnel, injection of environmental air into the vial during extraction, duration of use and storage, conditions of container storage (temperature, sun exposure, and others), the presentation of preservatives in the vial (5, 6). Drugs without preservative and drugs containing lipids are more prone to contamination (7). It was reported that the administration of contaminated MDVs, SDVs and ADXs with *Pseudomonas aeruginosa*, *Enterobacter cloacae*, *Candida albicans *and *Serratia marcescens *were resulted in several cases of bloodstream infection, bacterial meningitides, wound infection and death in the receiving patients (8-11). Therefore, the aim of this study was to investigate the magnitude and pattern of microbial contamination of MDVs and SDVs after multiple uses in three different hospitals in Tehran as well as ADXs prepared in the treatment room by nursing staff versus those prepared by the hospital pharmacist in the clean room.

## Experimental

*Sample collection*

Without prior warning, opened vials of MDVs and SDVs that were kept for reuse in the wards and ADXs prepared in the treatment room by nursing staff or those prepared by the hospital pharmacist in the clean room were collected and the related information such as the drug type, production date, expiration date, first day of vial›s opening, labeling of vials and storage conditions were recorded. Characteristics of samples were shown in Tables 1 and 2. Samples were transferred to the microbiology laboratory and analysis was started immediately. Prior to sampling, the vials were shaken vigorously and the gums were swabbed with70% ethyl alcohol.

**Table 1:** Distribution of the collected vials

**ward/Hospital**	**Sample type** ^a^	**Number of vials**
Imam Khomeini/infection ward	MDV-SDV	21
Children medical center/surgery ward	MDV-SDV	22
Shariati hospital/clean room	ADX	17
Imam Khomeini /infection ward	ADX	54
Children medical centre/surgery ward	ADX	38

^a^MDV: multiple-dose vial; SDV: single-dose vial; ADX: admixture

**Table 2:** Characteristics of the collected vialsa

**Sample**	**Number**	**Time in use**			
		**< 1 day**	**1-3 days**	**> 3 days**	**not known**
**MDVs**					
**Lidocaine 2%**	2	0	0	0	2
**Insulin NPH**	1	0	0	0	1
**SDVs**					
**KCl 15%**	10	1	2	1	6
**MgSO**_4_ ** 20%**	4	0	1	0	3
**MgSO**_4_ ** 50%**	6	1	0	2	3
**NaCl 0.9%**	3	0	0	0	3
**NaCl 5%**	2	1	0	0	1
**NaCl 0.45%**	1	0	0	0	1
**Phenytoin 250 mg/ 5 mL**	1	0	1	0	0
**Acyclovir 250mg**	1	0	1	0	0
**Methylprednisolone 500 mg/ 10 mL**	1	0	1	0	0
**Co-trimoxazole 480 mg/ 5 mL**	1	0	1	0	0
**N-acetylcysteine**	1	0	1	0	0
**Dextrose 50%**	4	1	2	0	1
**Amino acid10%**	3	0	2	0	1
**Calcium gluconate10%**	2	1	0	0	1
**ADXs**					
**Dextrose 5%**	45	NAb	NA	NA	NA
**Dextrose 10%**	7	NA	NA	NA	NA
**NaCl 0.9%**	27	NA	NA	NA	NA
**NaCl 0.45%**	19	NA	NA	NA	NA
**Dextrose 5%, NaCl 0.9%**	8	NA	NA	NA	NA
**Dextrose 0.33%, NaCl 0.3%**	3	NA	NA	NA	NA

^a^MDV: multiple dose vial; SDV: single dose vial; ADX: admixture.

^b^NA: not applicable; ADXs were used at the time of preparation.

*Microbiological diagnosis tests*

*Sterility testing of vials by using membrane filtration*

In order to increase the probability of microbial contamination detection of vials or ADXs, the total volume of remaining solutions were used. The culture media used in sterility tests were complied with the requirements of the growth promotion test of aerobes, anaerobes and fungi (12). Sterility test was performed following the method described by the United States Pharmacopeia (USP) (12) by using sterile membrane filter units (Millipore, USA) and 0.45 μm membranes with a diameter of approximately 47 mm. The remaining volume of each vial was divided in two parts and aseptically transferred directly into two separate membrane filter funnels. Then, each filter was washed with 100 mL of sterile saline to eliminate carryovers on the filter. The membranes were removed aseptically from the holders and immersed in 100 mL of CASO broth (Merck Co.) and Thioglycollate medium (Merck Co.) which were incubated at 25 ± 2.5 and 35 ± 2.5°C, respectively for at least 14 days. The vessels were visually inspected at 24-h intervals for the evidence of microbial growth. If microbial growth was found, the contaminant was purified and identified by Gram-staining, using suitable culture media and biochemical tests (13, 14).

*Microbial analysis of critical points*

Quantitative analysis of some critical points in the treatment rooms was performed by fingerprint method (nurse’s hand), swab technique (table of treatment room) and air sampling by S.T.A Biological Air Sampler (STA**-**303, New Brunswick Scientific) using CASO agar plates. The viable microbial counts were determined after 72 h of incubating the plates at 35 ± 2.5°C.

## Results and Discussion

*Sterility testing of vials*

The results of sterility testing of 43 vials (MDVs and SDVs) and 92 ADXs prepared in traditional treatment rooms and 17 ADXs prepared in clean room showed that only one of 92 ADXs prepared in the treatment room (1 L NaCl 0.45% plus 10 mL KCl 15% and 2.5 mL MgSO_4_) was contaminated with *Bacillus subtilis *and none of the samples prepared in clean room had microbial contamination.

At time of collecting samples, only 20/43 of MDVs and SDVs (46.5%) had the date of first day of vial’s opening; 5 vials were opened less than 1 day, 12 were opened between 1 to 3 days and 12 vials were opened beyond 3 days.

*Investigation of critical points*

The total microbial count and identified bacteria from the nurse’s hand, surfaces and air of treatment rooms are shown in Table 3. The total microbial count of different examined parts were in the range of 4-13 CFU/nurse’s hand, 3-10 CFU/400 cm^2^ of treatment surface and 9-12 CFU/m^3^of the treatment air.

**Table 3:** Isolated bacteria and total microbial counts of critical points in treatment rooms

**Microbial count**	**Source**	**Hospital**
**13 CFU/5 fingers**	Fingers of the nurse	Children medical center
**4 CFU/5 fingers**	Fingers of the nurse	Imam Khomeini hospital
**10 CFU/400 cm** ^2^	Table surface	Children medical center
**9 CFU/m** ^3^	Air	Children medical center
**12 CFU/m** ^3^	Air	Imam Khomeini hospital
**3 CFU/400 cm** ^2^	Table surface	Imam Khomeini hospital

ADX solutions were prepared routinely for injection to the patients by the nurse in the treatment room. This room had no standard conditions such as laminar airflow (LAF) hood, air filter and special clothes. Therefore, importance of environmental health such as use of a clean room has been emphasized in order to prepare sterile ADXs in hospitals (15). In accordance with the recommendation of CDC, when standard aseptic methods are used for preparing and keeping of ADXs, these solutions can be kept in the refrigerator for one week (16). It was reported that the administration of contaminated MDVs, SDVs and ADXs with *Pseudomonas aeruginosa*, *Enterobacter cloacae*, *Candida albicans *and *Serratia marcescens *resulted in several cases of septicemia, bacterial meningitides, wound infection and death in receiving patients (8-11). A study comparing the contamination of ADXs prepared under LAF hood with ADXs prepared by the nurse in nursing unit showed higher contamination rate in ward (10.9%) in comparison with ADX prepared under LAF hood (5.5%) (17). The rate of ADXs contamination was reported in range of 0-14.5% (3) and contamination of MDVs was reported between 0 and 27% (4).

In our study, none of all 43 opened MDVs and SDVs which were kept for multiple uses in the wards were culture-positive. The low microbial contamination of nurse’s hand, air and surfaces of the treatment room indicated that sanitation practices have been well established in the hospital wards and may be a reason for the low contamination rate of MDVs, SDVs and ADXs prepared in treatment rooms. Our results showed that although traditionally prepared ADXs in treatment room have low contamination rate (1.1%), they have higher contamination in comparison with ADX prepared in clean room (0%). It was demonstrated that the overall sterility assurance level for aseptically-produced products in class A controlled environment such as LAF hood is 10^-3^ (18). To reduce the risk of IV administration-related infections, attention to aseptic techniques such as disinfection of nurse’s hands and gums of the vials, the number of withdrawals made from the vial, injection of environmental air into the vial during the extraction, duration of use and storage, conditions of the container storage and the presentation of preservatives in the vial should be considered (5, 6). However, using clean room environment for preparation of ADXs could be the best strategy to reduce the contamination rate of ADX solutions.
